# Signaling of the Purinergic System in the Joint

**DOI:** 10.3389/fphar.2019.01591

**Published:** 2020-01-24

**Authors:** Carmen Corciulo, Bruce N. Cronstein

**Affiliations:** ^1^Division of Translational Medicine, Department of Medicine, NYU School of Medicine, New York, NY, United States; ^2^Krefting Research Centre—Department of Internal Medicine and Clinical Nutrition, Institute of Medicine, University of Gothenburg, Gothenburg, Sweden; ^3^Division of Rheumatology, Department of Medicine, NYU School of Medicine, New York, NY, United States

**Keywords:** adenosine, cartilage, bone, synovium, tendon, purine, adenosine triphosphate, adenosine diphosphate

## Abstract

The joint is a complex anatomical structure consisting of different tissues, each with a particular feature, playing together to give mobility and stability at the body. All the joints have a similar composition including cartilage for reducing the friction of the movement and protecting the underlying bone, a synovial membrane that produces synovial fluid to lubricate the joint, ligaments to limit joint movement, and tendons for the interaction with muscles. Direct or indirect damage of one or more of the tissues forming the joint is the foundation of different pathological conditions. Many molecular mechanisms are involved in maintaining the joint homeostasis as well as in triggering disease development. The molecular pathway activated by the purinergic system is one of them.The purinergic signaling defines a group of receptors and intermembrane channels activated by adenosine, adenosine diphosphate, adenosine 5’-triphosphate, uridine triphosphate, and uridine diphosphate. It has been largely described as a modulator of many physiological and pathological conditions including rheumatic diseases. Here we will give an overview of the purinergic system in the joint describing its expression and function in the synovium, cartilage, ligament, tendon, and bone with a therapeutic perspective.

## Anatomy and Function of the Joint

**Figure 1:**
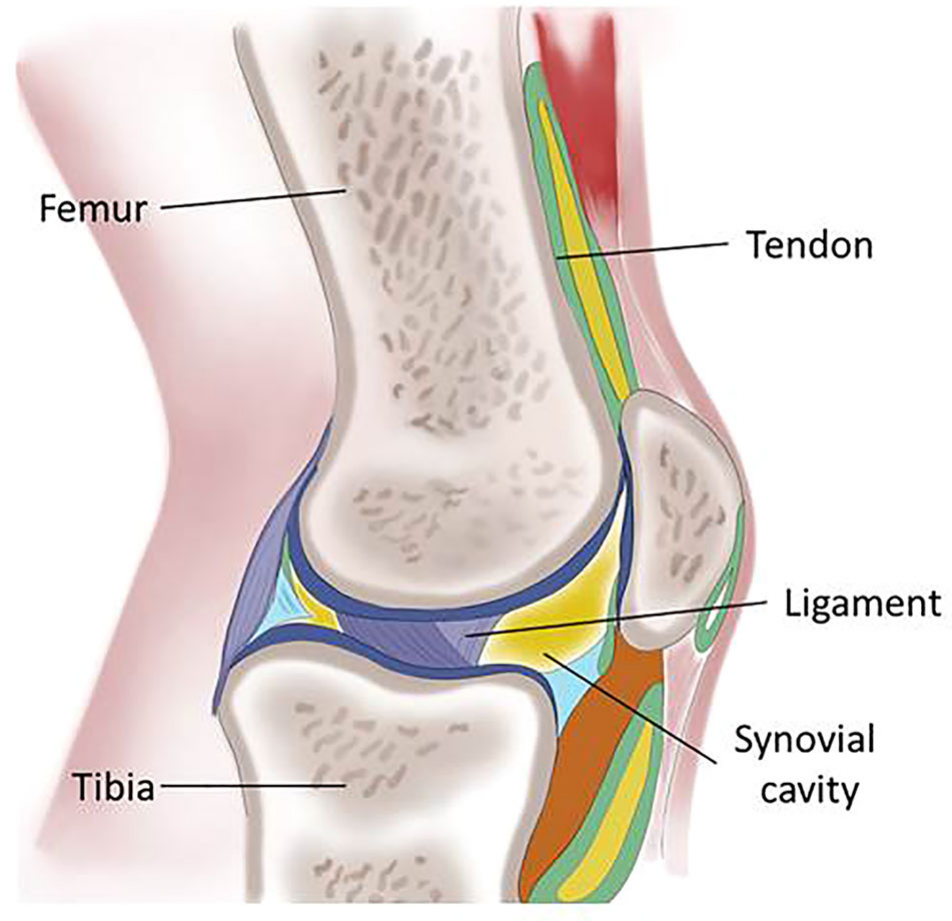
Schematic representation of the knee describing the main tissues of the articular joint: femur and tibia, the skeletal elements are maintained in position by the ligaments and connected to the muscles by the tendons; the synovial cavity is lined by the synovial membrane.

The joint, the structure around the potential space between bones, is a complex anatomical structure consisting of several different types of tissues that together, permit mobility and stability at the body (**Figure 1**). Joints located in different anatomical areas have their own structure which evolved to counteract the different intensities of mechanical loading and the need for fine movements.

A first joint classification is based on the features of the link between two bones: in the fibrous joint, there is no cavity between adjacent bones connected by fibrous tissue; in the diarthrotic joint movement can take place freely in various planes; the amphiarthrosis joint is slightly movable and ligaments connect the bones. The diarthrosis joints are surrounded by an articular capsule composed of connective tissue filled with synovial fluid allowing free movement and giving stability protecting from dislocation. Bones provide stability and support the muscle. The skeleton is kept in appropriate alignment by ligaments and connected to the muscle by tendons (Anderson and Bordoni, 2019).

Many pathological conditions directly or indirectly affect the joint such as osteoarthritis and rheumatoid arthritis (RA) and they may be due to aging, mechanical stress, or injury as in the case of ligament rupture, or to general inflammatory status as in rheumatoid arthritis (Lieberthal et al., 2015; Tracy et al., 2017).

Appropriate interactions between these different tissues are carried out at the cellular and molecular level by numerous molecules and molecular pathways activated and the purinergic system is one of them (Rumney et al., 2012; Cronstein and Sitkovsky, 2017).

In this review, we will describe the expression and function of the purinergic system in the joint.

## Purinergic System

The purinergic signaling is activated by the extracellular binding of adenosine, adenosine diphosphate (ADP), adenosine triphosphate (ATP), uridine triphosphate (UTP), and uridine diphosphate (UDP) to transmembrane receptors and ions channels. This system was characterized in the early 1990s with the description of four G-protein coupled (GPCR) adenosine receptors (P1), seven P2X ion channels activated by ATP, and eight P2Y G-protein coupled receptors, binding sites for ATP, ADP, UTP, and UDP (Menzies et al., 2017; Burnstock, 2017).

In the resting state the amount of ATP in the cytosol is between 1 mM and 5 mM whereas it is much lower in the extracellular space ranging in the nanomolar concentration (10–100 nM) (Bours et al., 2006). The extracellular nucleotides are released massively from the cytosol as a “danger” signal and their concentration increases during apoptosis, mechanical stress, e.g. stretch and shear stress, in hypoxic conditions and during infection. ATP can be released into the extracellular space by vesicular transport or through channels localized on the cell membrane, like Pannexin and Connexin channels, or it can reach the extracellular space after cell lysis (Di Virgilio et al., 2009). ATP and ADP bind and activate their own receptors: P2Y receptors, including eight subtypes of metabotropic receptors coupled to G-proteins, seven P2X hetero or homotrimers forming ion channels permeable to Na+, K+, and Ca2+ upon ATP binding (Abbracchio et al., 2006; Kaczmarek-Hajek et al., 2012; Sluyter, 2015). Prolonged activation of P2X receptors can lead to conformational changes in the structure of the trimers leading to higher ion permeability or to channel desensitization, an effect depending on the receptor subtype activated (Khakh et al., 1999; Saul et al., 2013). The functionality of P2Y receptors is instead regulated by the recruitment of β-arrestin (subtypes 1–4) which promotes inhibition of the interaction receptor G-protein (desensitization), receptor internalization or activates the signaling cascade. Which subtype of β-arrestin is recruited and the intensity of the interaction depends on the receptor-activated and on the ligand involved, ATP or UTP (Erb and Weisman, 2012).

**Figure 2:**
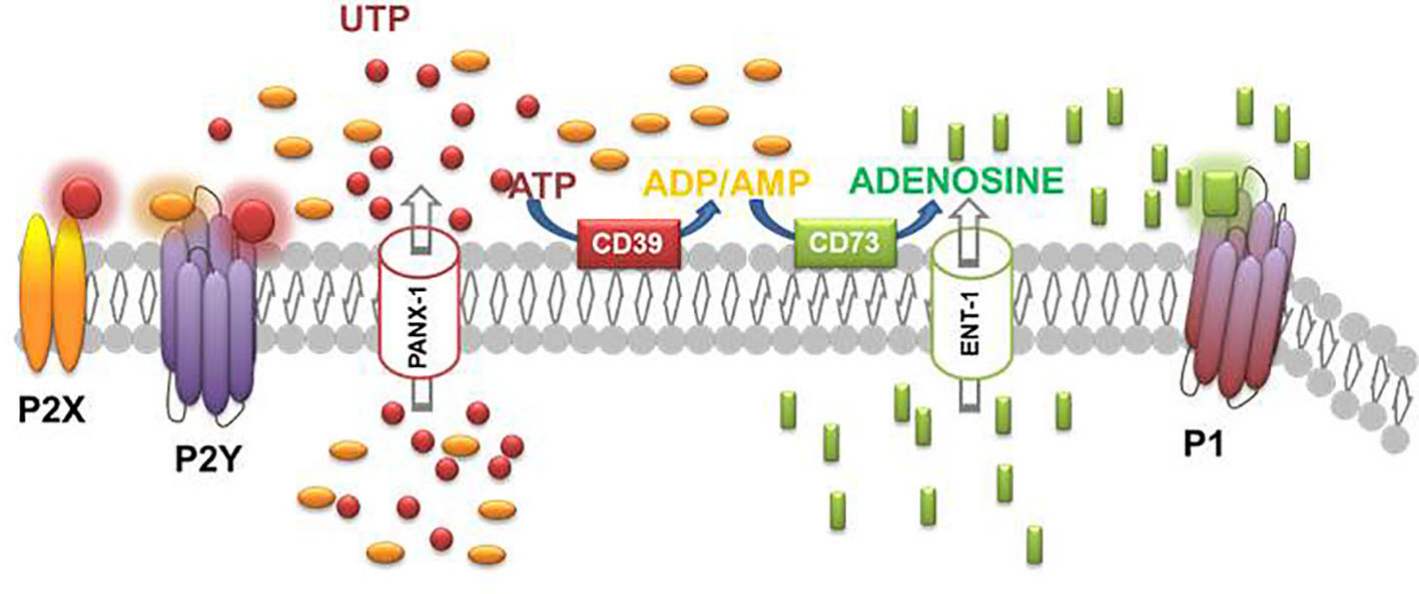
Schematic diagram illustrating the effectors, enzymes, channels, and receptors playing a role in the purinergic system.

ATP is also the source of adenosine. Adenosine is released into the extracellular space by most cells and tissues. The basal level of adenosine in extracellular fluids is roughly 100 nM and its concentration increases during periods of cell stress rising into the low micromolar range. This increase in concentration is the result of more adenosine released through the equilibrative nucleoside transporter (ENT) or, more importantly, release of adenine nucleotides which are hydrolyzed by a series of cell surface and soluble enzymes, including ectonucleoside triphosphate diphosphohydrolase 1 (E-NTPDase1; from ATP to ADP and AMP) and ecto-5`-nucleotidase, also known as CD73, that converts AMP into adenosine. Finally, adenosine in the extracellular space can be metabolized to inosine by adenosine deaminase (ADA) or taken up by cells and rephosphorylated by adenosine kinase (Hasko et al., 2018) (**Figure 2**).

There are four subtypes of adenosine receptors named A1, A2A, A2B, and A3 which are all members of the large family of G protein-coupled receptors (GPCRs). A1 and A3 receptors are coupled to Gi signal transduction proteins which inhibit adenylate cyclase; A2A and A2B receptors promote cAMP synthesis by coupling to Gs protein; A2BR is also coupled to Gq protein (Ryzhov et al., 2006). Adenosine binds A1 and A2A receptors with an affinity of 10–30 nM; the affinity for A3 receptor is roughly 1 uM and even higher for the A2BR (Fredholm et al., 2011). The downstream signaling receptor function is regulated by β-arrestin that, preventing interaction of the receptor with the G proteins, switches off the signaling and mediates internalization of the receptor (Verzijl and Ijzerman, 2011).

The purinergic system aroused interest in the rheumatology and orthopedic field for its direct effect on the joint and indirect effect on articular tissues mediated by modulation of the immune response.

In the next paragraphs, we will describe the direct effect of the purinergic system activation on the different joint tissues.

## The Purinergic System in the Joint

### Cartilage

#### P2 Receptors

P2 receptors were first identified in human chondrocytes in 1991 as mediators of prostaglandin E2 release (Caswell et al., 1991) and more recently there is a deep focus in the field on the role of ATP in mechanotransduction and cartilage homeostasis (Millward-Sadler et al., 2004; Knight et al., 2009; Garcia and Knight, 2010).

The activation of P2 receptors following mechanically induced ATP release or addition of exogenous ATP has an anabolic effect, with up-regulation in proteoglycan, collagen synthesis, and cell proliferation (Brown et al., 1997; Croucher et al., 2000; Picher et al., 2003; Millward-Sadler et al., 2004; Chowdhury and Knight, 2006). In contrast, it has also been reported that ATP stimulates proteoglycan breakdown and glycosaminoglycan release from bovine nasal cartilage explants and thus, may have a role in diseases that primarily involve the destruction of non-articular cartilage (Leong et al., 1990; Brown et al., 1997). Interestingly the opposite effect was measured when a similar experiment was performed on articular cartilage confirming that the heterogeneous results depend on the tissues, articular vs. non-articular cartilage, involved (Brown et al., 1997). In fact, bovine primary articular chondrocytes culture supplemented with ATP increases neocartilage collagen by 110% (Garcia and Knight, 2010).

In pathological conditions, high levels of ATP have been associated with cartilage degradation and pathological calcification. Mutation in the progressive ankyloses gene, ANK, leads to calcium pyrophosphate dihydrate crystal deposition (CPPD) in the joint and consequent cartilage degradation. Overexpression of ANK correlates with an increase of extracellular ATP (Rosenthal et al., 2013). Because of this preliminary evidence, Probenecid, a drug in common use for the treatment of gout and which blocks ATP release by Pannexin1 and ANK channels, is currently in clinical testing in CPPD patients (https://clinicaltrials.gov/ct2/show/NCT02243631) (Silverman et al., 2008).

The articular cartilage is subject and responsive to mechanical stimuli. Mechanical force on the extracellular matrix of the cartilage is transferred to the chondrocytes through integrins such as integrin B3, that transfer the stimuli at molecular level activating homeostatic mechanisms and compensating for the mechanical stress (Kudirka et al., 2007). Applying a 1 Hz mechanical force to a bovine chondrocyte culture in agarose produces a sevenfold increase of ATP release by the opening of the hemichannels (Garcia and Knight, 2010). Moreover, fluid flow shear stress increases secretion of ATP through connexin hemichannels promoting lubricin production (Graff et al., 2000; Millward-Sadler et al., 2004; Bao et al., 2004; Gomes et al., 2005; Ogawa et al., 2014). According to these studies it has been demonstrated that bovine and human chondrocytes express connexin 43 (Knight et al., 2009) and blockade of connexin hemichannels in chondrocyte culture reduces extracellular ATP and collagen II production (Schrobback et al., 2015). Moreover experiments performed on primary chondrocytes subjected to cyclic strain and mice subjected to treadmill running confirm that extracellular ATP is an important mediator of mechanotransduction which downregulates the expression of metalloproteinases MMP-1 and MMP-13 (He et al., 2016). Conversely, another research group demonstrated that prolonged cyclic axial compression of chondrocytes suppressed the extracellular ATP level and contributed to the destabilization of cartilage (Coleman et al., 2016). Human chondrocytes stimulated with a mechanical force of 0.33 Hz hyperpolarize through a mechanism mediated by ATP release and P2Y receptors activation. Interestingly, no change in membrane polarization was measured in OA chondrocytes (Millward-Sadler et al., 2004). Moreover we have demonstrated that incubation of murine chondrocytes with IL-1β decreases ATP release (Corciulo et al., 2017). All these results document the impairment of the ATP signaling during OA development and inflammation of the joint suggesting a constitutive activation of P2 receptors in chondrocytes is associated with cartilage protection and maintenance of the homeostasis of the extracellular matrix.

**Figure 3:**
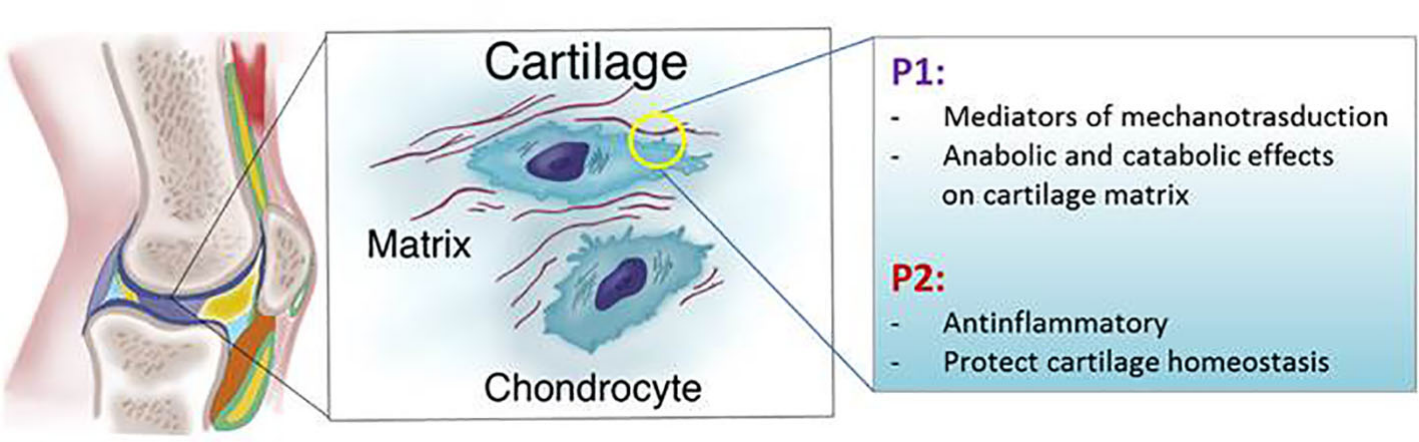
Schematic representation of P1 and P2 receptors functions in articular cartilage.

Because different studies have yielded different results it is not clear whether ATP released in physiological conditions into the extracellular space protects or mediates cartilage damage during mechanical stress. These contradictory findings suggest that the response to ATP may either be dose-dependent or that the heterogeneous response depends on the health status of the joint, on which cartilage (articular or non-articular) is involved and what kind of mechanical forces are applied to the tissues (**Figure 3**).

#### P1 Receptors

The presence of P1 receptors on chondrocytes was demonstrated in 1999 by Koolpe and colleagues in human articular chondrocytes (Koolpe et al., 1999) and the homeostatic role of adenosine in cartilage and chondrocytes has been described by our and other laboratories. All adenosine receptors are expressed in bovine, mouse, and human articular chondrocytes (Varani et al., 2008; Varani et al., 2008; Vincenzi et al., 2013). Adenosine is responsible for reduced NO production in equine cartilage explants incubated with LPS, an effect mediated by ligation of A2AR and confirmed by incubation of chondrocytes with adenosine deaminase or an A2AR antagonist. Moreover it has been documented that A2AR agonists increase cAMP in equine chondrocytes and reduce the Il-1β mediated TNF-α, IL-6, MMP-13, and NO production (Tesch et al., 2002; Campo et al., 2012). The depletion of adenosine by adenosine deaminase also increases the production of metalloproteinases, PGE2 and NO, shedding light on the homeostatic role of adenosine in healthy cartilage and suggesting a constitutive activation of its receptors (Tesch et al., 2002; Benton et al., 2002; Tesch et al., 2002; Mistry et al., 2006). In our laboratory we demonstrated that adenosine is an important homeostatic regulator of chondrocytes during inflammation. We showed that in chondrocytes and rat cartilage explants transcripts of CD73 and channels for adenosine and ATP release are down-regulated after IL-1β incubation as well as ATP and adenosine concentrations in the extracellular space. The replacement of adenosine in the intraarticular space has shown delayed osteoarthritis progression in a rat model of post-traumatic osteoarthritis through a molecular mechanism involving A2A adenosine receptor activation (Corciulo et al., 2017). We also demonstrated that mice lacking A2A adenosine receptors develop spontaneous bone and cartilage features of OA associated with reduced motor activity and Shkhyan and colleagues demonstrated that ablation of A3 receptors results in OA development in aged mice (Corciulo et al., 2017; Shkhyan et al., 2018) (**Figure 3**).

### Synovial Tissue

The synovial tissue is a two cell layers thick membrane containing mainly two kinds of cell populations: synovial tissue macrophages (type A cells) and fibroblast-like synoviocytes (type B cells) (Falconer et al., 2018). Infiltration of lymphocytes, mild in OA and extensive in RA, leads to tissue hyperplasia contributing to inflammation, neovascularization, cartilage degradation, and pain sensitization (Mor et al., 2005; Asif Amin et al., 2017; Belluzzi et al., 2019). Activated synovial fibroblasts are involved in cartilage damage, particularly in RA subjects, *via* production of IL-6, IL-8, and TNF-α.

#### P2 Receptors

ATP release into the synovial fluid is triggered by the hypoxic environment in the synovium and hypotonic nature of the synovial fluid typical of RA. When synoviocytes *in vitro* were challenged with a hypotonic shock they activated a mechanism of calcium-mediated ATP release leading to reduced cell viability and synovium membrane hyperplasia (Hu et al., 2017). A study in 28 OA patients demonstrated that ATP concentration in the synovial fluid positively correlates with pain experienced by the patients. Moreover ATP concentration decreased after hyaluronic acid treatment (Kumahashi et al., 2011). Similar results were also demonstrated in OA dogs (Torres et al., 2016).

Synoviocytes isolated from RA patients express P2X1, P2X2, P2X3, P2X4, P2X5, P2X7, P2Y1, P2Y2, P2Y4, P2Y6, P2Y11, P2Y12, P2Y13, P2Y14 receptors whereas in synovial cells from OA patients two membrane ion channels, P2X(1) and P2X(3) receptors, were present (Caporali et al., 2008; Varani et al., 2010). In the synovial membrane ATP also induces the production of brain-derived neurotrophic factor, a neuromodulator involved in nociceptive hypersensitivity, by activating P2X4 receptor (Klein et al., 2012). The same receptor promotes IL-1β release from SF isolated from OA patients (Fan et al., 2014). On the contrary, P2X1 receptors showed an anti-inflammatory effect reducing NF-kB activation and TNF-α release whilst P2X3 receptors mediated the opposite response (Varani et al., 2010).

**Figure 4:**
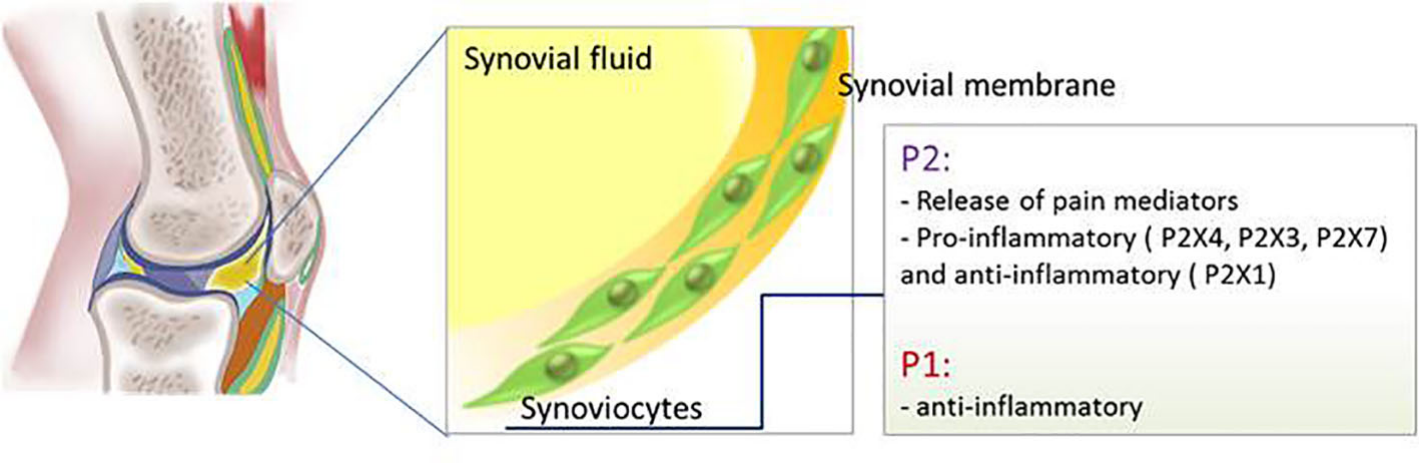
Schematic representation of P1 and P2 receptors functions in the synovial membrane.

P2X7 receptor expression was detected in inflamed synovial tissue in a rat arthritis model and inhibition of this receptor reduced articular inflammation and progressive bone destruction in a rat model of collagen-induced arthritis, an effect due to the attenuation of the leukocytes inflammatory response (Cronstein et al., 1993; McInnes et al., 2014). Despite the positive results in the pre-clinical study, antagonists for P2X7 receptor, CE-224,535 and AZD9056, did not show any efficacy when administrated orally to patients with rheumatoid arthritis (Montesinos et al., 2000; Montesinos et al., 2003) (**Figure 4**).

#### P1 Receptors

mRNA of all adenosine receptors are expressed in human SF where they exert an anti-inflammatory function (**Figure 4**). In particular adenosine inhibits the pro-inflammatory pathway activated by NF-kB through binding to A2AR and A3R (Varani et al., 2010) whereas it has been shown that the anti-inflammatory effect of the electromagnetic field on synovial fibroblasts is mediated by A1R and A2AR upregulation (De Mattei et al., 2009).

Another evidence of the anti-inflammatory role of adenosine comes from the measurement of ADA levels in the synovial fluid, higher in the RA patients compared to the OA patients, and correlating with the disease severity (Sari et al., 2003). Nevertheless treatment of RA synoviocytes with 2-chloroadenosine (2-CADO), an adenosine analog resistant to adenosine deaminase, induces cell apoptosis independent from adenosine receptors activation. Interestingly adenosine does not have the same of 2-CADO effect on cell apoptosis (Koshiba et al., 2002) pointing out that regulation of adenosine amount in the extracellular space is fundamental to avoid cell toxicity. It is a matter of fact that patients with adenosine deaminase 2 deficiency present vascular symptoms and rheumatic inflammatory features (Hashem et al., 2017).

Fibroblasts like synoviocytes isolated from RA patients express all adenosine receptors. In RA A3R is expressed significantly more than the other receptors while A2AR is less expressed (Stamp et al., 2012). Targeting A3R to promoting activation of anti-inflammatory mechanisms has been proposed since the A3R agonist, CF101, showed efficacy in a pre-clinical RA model and in RA patients reduces their symptoms and signs (Silverman et al., 2008; Bar-Yehuda et al., 2009).

Adenosine exerts an indirect role of joint protection by modulating the pro-inflammatory NF-kB molecular signaling in immune cells. Increased expression of A2AR and A3R adenosine receptors has been measured in white cells of RA and in ankylosing spondylitis patients, an effect that decreased after anti TNF-alpha treatment, consistent with the known effect of TNF-alpha on the expression of adenosine A2AR *via* NF-kB activation (Varani et al., 2009; Varani et al., 2011; Ravani et al., 2017). Moreover, the effect of low dose, weekly methotrexate (MTX), the gold standard treatment for RA, is also mediated by increased released of adenosine and activation of A2AR and A3R. It is well established that MTX induces the release of adenosine and reduces inflammation in an adenosine-dependent manner (Cronstein et al., 1993). Initially, it was demonstrated that only non-selective antagonists for adenosine receptors were able to inhibit MTX anti-inflammatory effect consistent with the involvement of multiple receptors (Montesinos et al., 2000). Further studies suggested that low doses of MTX, comparable to the doses used in RA patients, were able to activate A2AR and A3R (in the air pouch model) and only A2AR in a model of peritoneal inflammation (Montesinos et al., 2003; Montesinos et al., 2006).

### Bone

Bone is a dynamic tissue subjected to continuous remodeling, adjusting to environmental changes and responding to inflammation, hormonal alterations, and mechanical injury in pathological conditions (Rowe and Sharma, 2019). Osteoblasts, osteoclasts, and osteocytes are the principal cell types that interact and cooperate at the molecular level to orchestrate bone remodeling (Olsen et al., 2000). Osteoclasts are responsible for bone resorption and formation of resorption lacuna. They are differentiated from hematopoietic cells by a molecular mechanism controlled mainly by two cytokines, macrophage colony-stimulating factor M-CSF and the ligand for the receptor activator of nuclear factor kB (RANKL) (Kim and Kim, 2016). Osteoclasts polarize when they are in contact with a mineralized matrix forming specific domains including the sealing zone, the ruffled border, and the functional secretory domain, all important for creating a compartment for bone reabsorption (Martin and Sims, 2005; Crockett et al., 2011). Osteoclasts synthesize MMP-2, 4, 6, and 7 important for osteoblasts recruitment and activation (Garimella et al., 2008). Osteoblasts, instead, differentiate from mesenchymal precursors in the bone marrow (Ponte et al., 2007; Uccelli et al., 2008; Garg et al., 2017). During bone formation osteoblast precursors interact with the bone surface to generate a calcified matrix (Marie et al., 2014). Osteocytes are embedded in the bone matrix and are connected to each other by long dendritic cell extensions through a network made by canaliculi through which they sense changes in mechanical loading and regulate the function of osteoblasts and osteoclasts (Hemmatian et al., 2017).

**Figure 5:**
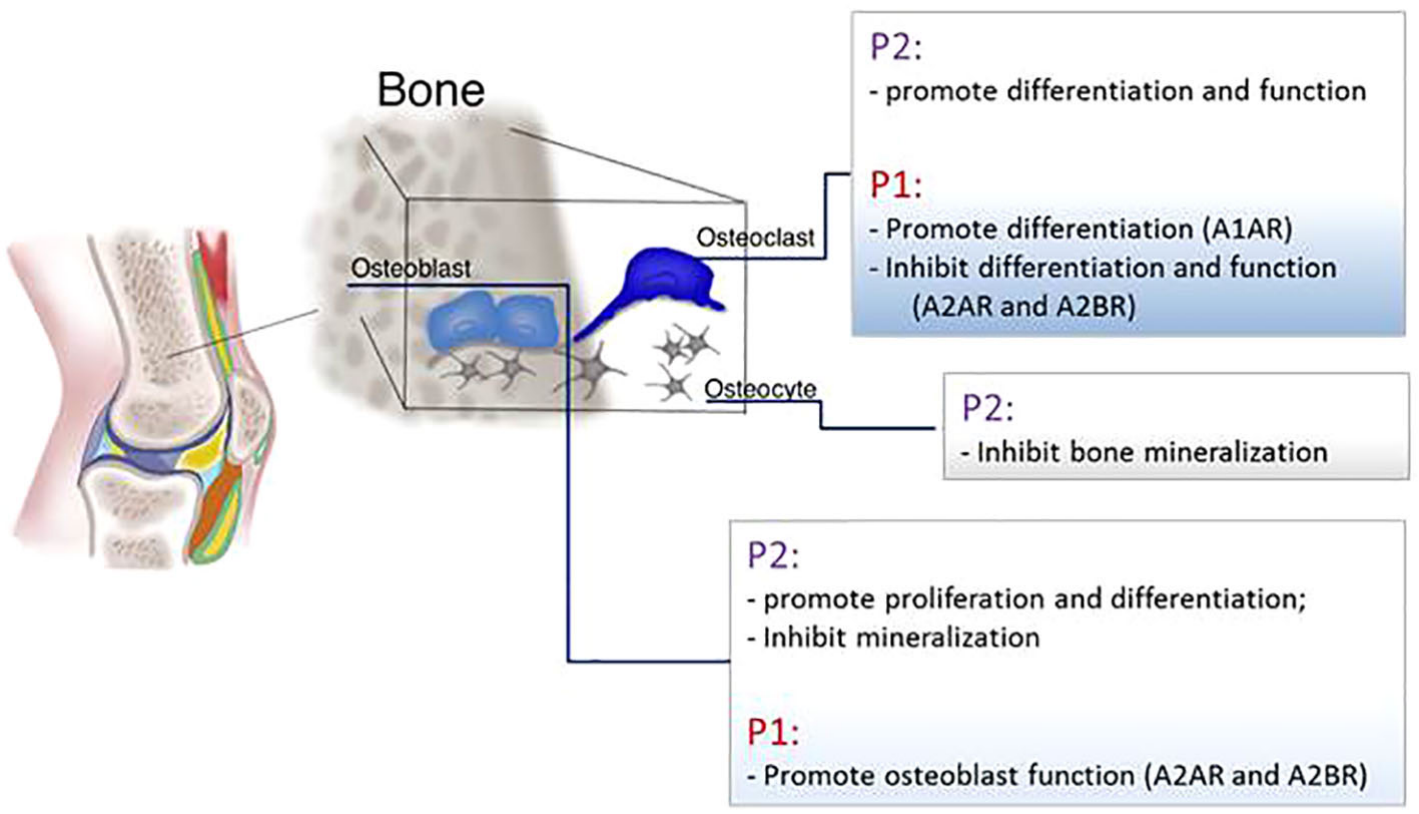
Schematic representation of P1 and P2 receptors function in the main bone cells: osteoblasts, osteoclasts, and osteocytes.

The regulation of the bone homeostasis is heavily dependent on the purinergic system (**Figure 5**).

#### P2 Receptors

ATP release and P2 receptor signaling are key modulators of skeletal development and homeostasis. Pannexin and connexin channels, P2X and P2Y receptors were detected in osteoblasts, osteoclasts, and osteocytes (Orriss et al., 2006; Plotkin et al., 2017). In osteoblasts mRNA for P2Y and P2X receptors were detected and it has been shown that their expression, as well as the amount of ATP released, depends on cellular differentiation status since osteoblasts’ responsiveness to nucleotides increase in parallel with the cell differentiation and an increase of P2Y2 and P2X4 receptors was detected in mature osteoblasts compared to immature cells (Bowler et al., 1995; Maier et al., 1997; Hoebertz et al., 2000; Ihara et al., 2005; Orriss et al., 2006; Orriss et al., 2009; Orriss et al., 2012; Syberg et al., 2012). ATP release in osteoblasts is also linked to cell proliferation since it has been shown that activation of P2X5 receptor promotes DNA synthesis and P2Y2 increases the proliferation rate (Suzuki et al., 1993; Nakamura et al., 2000; Katz et al., 2008; Ayala-Pena et al., 2013). Also P2X7 receptor stimulation enhances mineralization of osteoblasts; defective osteogenesis in P2X7 KO mice leads to wider calvarial sutures (Panupinthu et al., 2007; Panupinthu et al., 2008; Manaka et al., 2015). In human osteoblasts and in rat osteosarcoma cell line, P2 receptors mediate intercellular propagation of the calcium wave (Jorgensen et al., 1997; Jorgensen et al., 2000). Moreover the calcium wave can propagate *in vitro* between osteoblasts and osteoclasts in a co-culture system, a mechanism involving the activation of the P2X7 receptor (Jorgensen et al., 2002). The effect on bone mineralization is instead controversial. Mechanical forces promote ATP release followed by RUNX2 activation in a human osteoblastic cell line (Costessi et al., 2005) but low concentrations of ATP inhibit mineralization by binding P2Y2 receptor and mice lacking this receptor show an increased bone volume (Orriss et al., 2007; Orriss et al., 2017). Clopidogrel, an inhibitor of platelet aggregation used as a therapy for secondary prevention of the stroke, is an antagonist of P2Y12 receptor and *in vitro* was able to inhibit murine osteoblast proliferation and viability and to reduce osteoblasts’ ability to form bone nodules. Moreover, *in vivo*, clopidogrel reduces trabecular bone in femur and tibia of ovariectomized mice (Syberg et al., 2012). Nonetheless, in patients clopidogrel does not increase fracture risk (Jorgensen et al., 2017).

P2 receptors are also expressed in bone resorption cells (Burnstock et al., 2013). Through P2 receptors on osteoclasts, ATP induces a non-selective cation current (through P2X receptor) and the release of calcium from intracellular stores (P2Y receptors). The incubation of osteoclasts with suramin, a P2 receptor antagonist, blocks the generation of calcium waves (Wiebe et al., 1999). The P2Y12 receptor plays an important role in promoting osteoclast differentiation and activity. In models of arthritis P2Y12KO mice have increased trabecular bone and therefore are protected from osteopenia induced by arthritis, tumor growth in bone, and by ovariectomy (Su et al., 2012). Also *in vitro*, the active metabolite of Clopidogrel, a P2Y12 receptor antagonist, inhibits osteoclasts differentiation (Mediero et al., 2016). P2Y6 receptor has been associated to osteoclasts apoptosis, promotes NF-kB activation and osteoclast activity (Korcok et al., 2005; Lee et al., 2013). P2Y14, also, regulates osteoclast precursor differentiation by inducing RANKL (Lee et al., 2013). An extensive scientific literature is dedicated to exploring the effect of P2X7 receptor on bone resorption. Two clinical studies involving respectively 506 and 1,764 post-menopausal women, demonstrated that polymorphisms in the gene coding for the P2X7 receptors that diminish the binding of ATP to the receptors, contribute to the increased risk of lumbar spine fractures (Ohlendorff et al., 2007; Gartland et al., 2012). It is generally accepted that the P2X7 receptor is implicated in the generation of multinucleated giant cells and the *in vitro* administration of P2X7 receptor antagonists to human osteoclast precursors inhibits osteoclast differentiation (Gartland et al., 2003; Agrawal et al., 2010). Gartland et al. demonstrated that mice lacking P2X7 receptor do not exhibit important skeletal alterations whereas Ke et al. measured a reduction in cortical bone of the femur and an increase of the bone resorption in the tibia. Interestingly, both groups reported that osteoclast precursors isolated from these mice are still able to activate the process of cell fusion indicating an alternative path to differentiation that does not involve the P2X7 receptor (Gartland et al., 2003; Ke et al., 2003).

ATP is also released by osteocytes after mechanical stimulus and nucleotide stimulation with the effect of inhibiting bone mineralization in the surrounding area allowing the formation of lacunae (Genetos et al., 2007; Hajjawi et al., 2014; Kringelbach et al., 2014). Osteocyte apoptosis is a mechanism required for bone remodeling and precedes bone resorption; late-stage osteocytes produce RANKL in order to activate osteoclast differentiation. ATP plays a role in inducing osteocyte apoptosis through activation of P2X7 receptor in the intracortical area (Cheung et al., 2016).

In osteoblasts, UTP, but not UDP, increases ALP activity and expression of the osteogenic proteins BMP-2, 4, and 5 (Ayala-Pena et al., 2013). It has been shown that UTP and UDP facilitate the osteogenic differentiation of human bone marrow cells by P2Y6 receptor ligation (Noronha-Matos et al., 2012). At the same time, UDP through P2Y6 receptor stimulates the formation of osteoclasts from precursor cells and enhances their reabsorptive functionality. As a result of P2Y6 receptor role, mice lacking this receptor display more cortical bone (Orriss et al., 2011).

#### P1 Receptors

Patients with mutations in the ADA gene and the consequent increase in adenosine availability, present radiological bone defect including scapular and ribs changes, alteration due to imbalance between osteoclasts and osteoblasts functions resulting in low bone formation (Cederbaum et al., 1976; Sauer et al., 2009; Manson et al., 2013). mRNA of CD39 and CD73 is expressed in precursor and mature osteoclasts (He et al., 2013) and mice lacking CD73 have osteopenia associated with a higher number of osteoclasts compared to control mice and present a delay in bone regeneration due to an impairment in osteoblast activity (Takedachi et al., 2012; Bradaschia-Correa et al., 2017). Administration of Dipyridamole (440 mg/day), a drug that blocks adenosine re-uptake increasing its extracellular availability, was tested on RA patients and it has been shown that the drug does not raise blood purine levels and does not change patients symptoms (Forrest et al., 2006; Forrest et al., 2006). Instead, local administration of Dipyridamole by using 3D printed scaffolds was able to regenerate bone defects in mice and rabbits (Ishack et al., 2017; Conesa-Buendia et al., 2019; Witek et al., 2019; Lopez et al., 2019). These different outcomes could be explained by the rapid metabolism of adenosine in the bloodstream an effect avoided by the continuous and sustained local release mediated by the scaffold implant.

Adenosine promotes bone formation and resorption by activating different molecular pathways depending on which adenosine receptor is activated. Adult A1R KO mice show increased trabecular and cortical bone density compared to WT mice even if no difference in osteoblast number or osteoblast morphology has been measured (Kara et al., 2010). *In vitro*, the incubation of murine bone marrow and human osteoclasts precursors with A1R antagonists decreases their differentiation (Kara et al., 2010). For its effect, antagonism of A1R was studied in a post-ovariectomy osteoporosis mouse model and it has been shown that A1R blockade prevents bone loss primarily by inhibiting osteoclast differentiation without affecting osteoblast function and number (Kara et al., 2010). At the molecular level, the effect of A1R activation on osteoclasts is mediated by NF-kB and TRAF6 signaling leading to RANK activation (He and Cronstein, 2012).

With regard to the role of A2AR, *in vitro* studies gave contrasting results depending on what kind of osteoclast precursor has been used. Pellegatti and colleagues demonstrated that A2AR promotes osteoclasts differentiation from peripheral blood mononuclear cells facilitating osteoclast fusion (Pellegatti et al., 2011). In contrast, Mediero and colleagues demonstrated that activation of A2AR and cAMP activation reduces the number of differentiated osteoclasts isolated from murine bone marrow. These results were confirmed by the observation *in vivo* of A2ARKO mice showing increased osteoclast number and osteopenia (Mediero et al., 2015).

A2AR exerts also an indirect inhibitory effect on osteoclasts by blocking the production of pro-inflammatory cytokines like TNF-α and IL-1β leading to diminished RANKL production in an inflammatory setting (Kim et al., 2005; Chan and Cronstein, 2010; Bitto et al., 2011). Indeed, methotrexate treatment diminishes wear particle-induced osteolysis *via* adenosine ligation of A2AR (Mediero et al., 2015) and A2AR agonists reduce bone resorption and disease progression in an animal model of type II collagen-induced arthritis (Bitto et al., 2011; Mazzon et al., 2011). Similarly, the administration of dipyridamole, an agent that enhances extracellular adenosine by blocking ENT1-mediated adenosine uptake, promotes bone regeneration (Mediero et al., 2015; Mediero et al., 2016; Ishack et al., 2017). In our previous study we observed an osteopenic phenotype in A2BRKO mice and Carroll and colleagues demonstrated delayed fracture healing in this mouse strain compared to the WT. This phenotype has been ascribed to the inability of the murine A2BRKO cells to differentiate into osteoblasts and to enhanced osteoclast differentiation (Carroll et al., 2012; Corciulo et al., 2016). Similarly, human bone marrow stimulated with A2BR agonist diminishes osteoclasts differentiation (He et al., 2013).

No direct effect of A3R activation on bone has been reported. Moreover, neither the A3R activation nor blockade affects osteoclast differentiation *in vitro* (He et al., 2013). Instead, the effect of A3R on bone has been studied has a consequence of its anti-inflammatory function. In an arthritis rat model, the treatment with an A3R agonist reduces inflammation, bone destruction, and the number of osteoclasts in bone surface (Rath-Wolfson et al., 2006).

### Ligaments and Tendons

Ligaments and tendons in the joint support and transmit mechanical loading to the musculoskeletal system by forming a dynamic connection between the skeletal bones and by linking muscle to bone. Injury or diseases of the connective tissue impair the mechanical function of ligaments and tendons mainly by reducing or changing their collagen composition (Zitnay and Weiss, 2018).

One of the diseases affecting the ligament is called ossification of the posterior longitudinal ligament spinal (OPLL) ligament due to ectopic bone formation of the spinal ligaments compressing the nerve root and causing neurologic problems. High levels of P2Y1 receptor expression and ATP release are responsible for osteoblastic differentiation of the cervical spinal ligament in patients affected by OPLL (Tanaka et al., 2011).

The role of the purinergic system in ligament function and structure has been largely studied in the periodontal tissues. In the periodontal ligament, P2Y4 and P2Y6 receptors are the most abundant P2Y receptors through which gravity released ATP induces phosphorylation of ERK and consequently collagen I and OPG release, essential for remodeling of the alveolar bone (Ito et al., 2014). In the human periodontal ligaments, the compression force promotes ATP release from the connexin 43 channel. P2Y receptor activation stimulates osteopontin and RANKL release contributing to the periodontal bone remodeling (Luckprom et al., 2010; Luckprom et al., 2011).

*In vitro*, elevated levels of ATP, typical in inflamed areas, increase apoptosis and caspase 3/7 expression of the periodontal ligament (Kawase et al., 2007). Periodontal ligaments, isolated from healthy volunteers, treated with the pro-inflammatory stimuli TNF-α or IL-1β showed a decreased expression of P2X7 receptor, the receptor associated with matrix mineralization and osteoblast differentiation (Xu et al., 2019).

**Figure 6:**
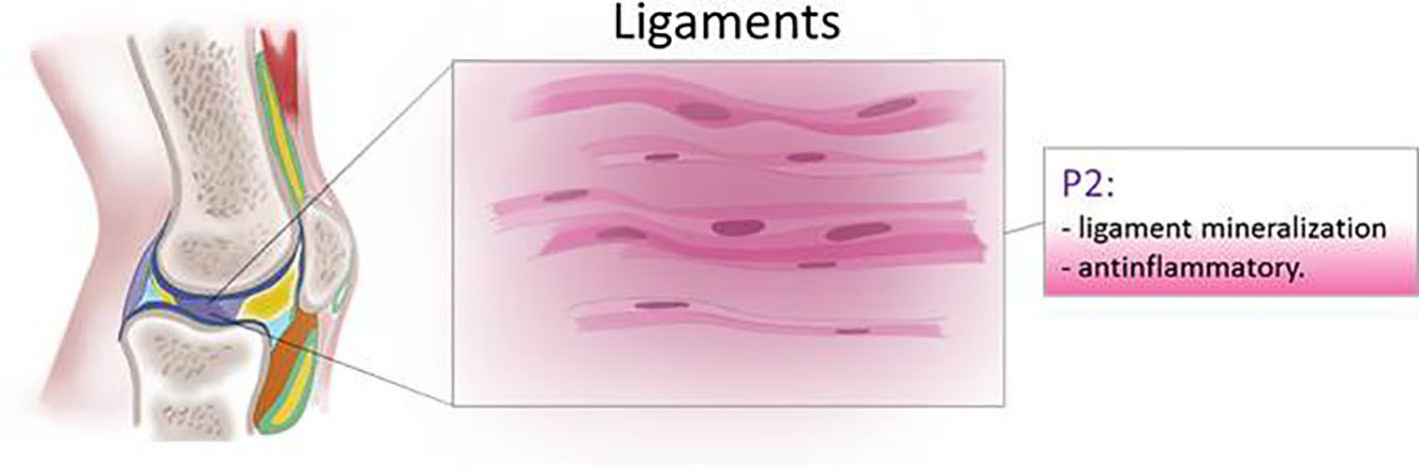
Schematic representation of P2 receptor functions in ligaments.

Human tendon cells subjected to mechanical stretch release IL-1β, COX-2, and MMP-3 and ATP which has been proposed as a trigger of negative feedback to limit activation of the inflammatory pathway (Tsuzaki et al., 2003) (**Figure 6**).

## Conclusion

In summary, extracellular purine nucleotides and their receptors are well established regulators of the viability and function of the tissues in the joint, playing an important role in maintaining homeostasis of the joint tissue cells. Stress and inflammation regulate the release of adenine and uridine nucleotides into the extracellular space as well as the enzymes that metabolize them. These interactions offer potential targets for interventions in diseases and conditions of the musculoskeletal system. Many attempts were made to target purine receptors to counteract joint damage in different rheumatic diseases, in human patients and in murine animal models, suggesting the efficacy of these drugs in counteracting inflammation. Nevertheless, since both P1 and P2 receptors are expressed on many different cells and organs it is likely that on-target toxicities will limit targeting of these receptors by systemic administration of agonists/antagonists. Local drug delivery of highly selective agonists and antagonists offer a promising way to make use of the beneficial effects of these agents.

## Author Contributions

CC: Literature search and drafting the manuscript. BC: revising and completion of the final work.

## Funding

This work was supported by the US National Institute of Health -NIAMS (R01AR068593) and the NYU- CTSI (U54TR001445).

## Conflict of Interest

CC and BC have a patent for the use of adenosine and A2AR agonists for the treatment of OA. BC and CC are co-founders and own stocks in Regenosine Inc.
